# Round the Hospitals

**Published:** 1918-01-19

**Authors:** 


### ROUND THE HOSPITALS.

On December 9 and 16, 1916, pages 210 and 225 respectively, we published the increases given in the nurses' salaries, and other interesting particulars in regard to emoluments of the nursing staff of the London and St. Thomas's Hospitals. To-day we have the pleasure to give a revised scale of pay for probationers at St. Bartholomew's Hospital. In order to make this information as useful and interesting as possible we have grouped these three hospitals in a table, setting out the figures, which is appended:—

*Probationers' Revised Scales of Pay.*

| Nurses in Training | St. Bartholomew's Hospital, Nov. 1, 1917. | St. Thomas' Hospital, Dec. 16, 1916 | | London Hospital, Dec. 9, 1916. |
|---|---|---|---|---|
| | Salaries Increased from | Salaries Classes I. | II. | Salaries Increased from |
| 1st year ... | £8 to £14 | — | £10 | £12 to £17 |
| 2nd year ... | £12 to £18 | £22 | £20 | £20 to £24 |
| 3rd year ... | £20 to £22 | £24 | £22 | £24, £25, or £27 to £30 for Staff Nurses. From £30 to £35 for Private Staff Nurses, rising by £5 to £50 |
| 4th year ... | £30 (no change) | £35 first 6 months, £40 second 6 months | £24 | |
| Those who remain a 5th year ... | £32 10*s*. | | | |
| Those who remain a 6th year ... | £35 | | | |

(1) At **St. Bartholomew's Hospital** the sisters start at £65, rising by £6 10s. after every completed five years' service to £84 10s. The out-patient sister starts at £71 10s., rising by the same increments to £84 10s.

(2) At **St. Thomas's Hospital** ward sisters commence at £44 per annum, rising to £54 the second year and £64 the third year. There are a number of special sisters' posts at St. Thomas's Hospital. Thus the sister massage is paid £150 a year, sister tutor £100 to £150, sister P.T. School £80 to £90, Sister Mary (lying-in ward) £80, the Home sister £70 to £90, sister out-patients £70, and sister in charge of *x*-ray department £70 to £80, in addition to full board, lodging, and laundry.

(3) It cannot fail to add to the interest to state that the **London Hospital private staff nurses,** who were formerly paid from £24 to £27 per annum, have all commenced their fourth year with a salary of £30, rising to £50 by increases of £5 a year. At the London Hospital ward sisters since December 1916 start at a salary of £40, and rise to £50 by £5 a year.

Trained nurses have been miserably underpaid in the past, and we hope that the above increases merely represent a beginning in the right direction. Will other important hospitals kindly send us figures and information showing the recent increases in salaries and emoluments?

Miss E. M. Cummins, matron of the Royal Infirmary, Liverpool, has been appointed a member of the Council of the College of Nursing. The College will require for its future development the continuous co-operation of the best brains and widest experience which the nursing profession contains. Miss Cummins' experience extends from her training-school in London to Bristol, from Bristol to the North of England, and thence to Liverpool, where she has exercised progressive influence for good and has maintained the high standards, the foundations of which were laid by her predecessors, who include one at least of the Queens of nursing. She has had wide and continuous experience in all fields of nursing, especially of its educational and administrative sides, and her selection is important at the present stage of development of the College, and full of promise for the future. The first election to the College Council should add trained nurses with experience, administrative gifts, and educational attainments to its counsels.

Records of courage and bravery in the face of danger are increasingly apparent among the staff of nurses engaged in war work, and it is pleasant to be able to record equal bravery and grit on the part of a private soldier in fighting for the life of a shipwrecked nurse. His Majesty the King is prompt to mark with his approval the display of gallantry and courage by trained nurses in the performance of their duties, the latest instance being the award of the Military Medal to Sister Mary Gladys Connie Foley, R.R.C., Q.A.I.M.N.S., and to Sister Mabel Jennings, A.R.R.C., T.F.N.S., who exhibited bravery and initiative when a casualty station was heavily shelled. The *London Gazette* of the 11th instant announces that the King has conferred the decoration of the Albert Medal on Private Samuel Arthur Bodsworth, R.A.M.C., in that he, on April 10, 1917, when H.M. hospital ship *Salta* was sunk in Havre Roads, remained in a swamped boat of the *Salta* instead of allowing himself to be rescued, in order to save a hospital sister who had become unconscious, around whose body he finally succeeded in placing a line, by means of which she was hauled on board H.M.S. *Druid*, which came to the rescue. Private Bodsworth incurred very considerable risk on account of the rough sea which prevailed at the time.

The City of London was reminded of Hun brutality and murder on the 17th instant, when the Lord Mayor, Lord Blyth, and other members of the British Ambulance Committee inspected outside the Mansion House a shell-shattered ambulance from Verdun, which remained on view for the citizens' inspection during the day. In this connection we cordially support the Dean of Manchester's call in the *Times* of the 14th instant for the exhibition by Englishmen of moral reprobation and invincible resolve by the calm, dis-

**ROUND THE HOSPITALS—(*continued*).**

passionate recital of Germany's crimes, up and down the country throughout every district, town and city, once or twice or oftener during each year the war continues. Dr. Welldon maintains such action would be the most powerful of all influences in stamping upon the mind and soul of Great Britain and the Allied nations the immutable conviction, that the war, which they are called to wage at an untold cost of suffering, is a war for all that is high and holy in the civilisation of the world. Dr. Welldon's point is that it is not implacable hatred but invincible resolve that is the spirit essential to our victory in the war.

Buried treasure used to be a newspaper heading which attracted all eyes, and excited a general desire on the part of the reader to stake a little money in any adventure advertised to proceed to that portion of the ocean where the treasure was supposed to lie, with a view to recovering it. The war has given rise to an exhibition of the attractive force of hidden treasure, which has been and is being exploited with marked success on behalf of Red Cross sales. Sir James Barrie, as the chairman of the Books and MSS. Committee, wrote to the *Times* asking readers to present treasures from their bookshelves for the forthcoming Red Cross sale at Christie's. Mr. Sydney Morse, who is chairman of the Silver Committee, followed this up by praising Sir James Barrie in words, and then conferred upon him the greater compliment of asking readers of the *Times* to present his Committee, for the same good cause, with their hidden treasures of gold, silver, and precious stones, which are too often locked away in safe or strong-room, to be seldom used or seen by the owners. Hidden treasure is not a possession which patriotic English men or women can cling to in existing circumstances. The owners of such rarely used and may be really valuable goods could by their means, through Messrs. Christie, bring support and comfort to many of those who are standing in the forefront of the battle for their country's honour. Just as buried treasures attracted public subscriptions to dubious enterprises, so previous Red Cross sales at Christie's prove that hidden treasures do, in fact, fetch prices which a short time back would have been considered impossible. In this connection, for the consideration of women only, we may relate an experience which has brought great pleasure and some cash to empty purses. Most of us have accumulated in drawers and other places various articles, sometimes purchased on a holiday, sometimes bought in a hurry, sometimes derived we know not whence, which lie forgotten until one day we are moved to turn the drawers out, and then the difficulty has been to decide what to do with the discoveries thus made. Might we suggest that every woman who reads this paragraph should turn to, empty her drawers and other receptacles, and see what she can discover and offer for sale, sending her gifts to Mr. Sydney Morse at 20 King Street, St. James's, S.W. 1, not later than February 14 next. One never knows one's luck. In searching for something for the Red Cross sales, one might find buried treasure too.

(*Continued on p.* 340.)

**ROUND THE HOSPITALS**—(*continued*).

MISS KATHLEEN STEWART, we regret to have to announce, has, after four years' faithful and most successful service as matron to the County Hospital, York, been forced to the conclusion by a letter received from the Committee that she had no course open to her, as a self-respecting woman, but to resign. The announcement of Miss Stewart's resignation has already caused strong feeling in York, we understand, where it is well known and admitted that the matron, her assistant matrons and sisters have worked loyally together, have pulled up the discipline and work in this hospital (which had previously lacked both), and until some year ago all went well to the advantage of everybody. It remains to be seen whether there is yet sufficient public interest taken in this hospital by the Governors and residents in York to institute an inquiry, and provide that the old bad state of affairs shall not be re-introduced into the management of the York County Hospital. The *Yorkshire Post* publishes a letter from Mrs. (?) Edith Milner, of Heyworth Hall House, York, in which she declares that

"universal regret is expressed on realising that Miss Stewart, the admirable matron of the York County Hospital, has sent in her resignation, which has been accepted. Why? will naturally be asked. During four years she has done her work so wisely and well, and brought the organisation to a high state of efficiency, she has had the additional responsibility of a large hut and three wards set apart for wounded soldiers, and she has carried out this work in a truly excellent manner. It is not too much to say the men worship her. The matron's lightest wish is law. As one who almost daily visits the hospital, as well as the other military hospitals, I can testify to her wonderful work and influence." She adds, "when a person of considerable influence asked why the matron was leaving, he was curtly told 'it was inevitable. No doubt York's loss will be a truly great gain for some other hospital.' But why should any change be made at this critical time?"

Mrs. (?) Milner ends her letter with the words "a dangerous expedient." Won't the Archbishop, or the Mayor, or the representatives of the working men in York City, or all three together, move, and insist on knowing exactly, why the York County Hospital should again become the home of crises, which every well and efficiently administered voluntary hospital is free from? What have the Board to say? What has the Committee, responsible, to urge in favour of the danger of a repetition of the unenviable reputation formerly enjoyed by this County Hospital?

---

WHEN visiting France we were much struck by the improved methods in cooking which some of our men had learned from association with their French comrades. We have been forced to use chilled meat to a much larger extent than usual in this country, and the latest intimation is that the trade are to be invited by the Government to co-operate with the Board of Agriculture and Fisheries in the matter of fish-freezing. Our experience leads us to wonder how many matrons, housewives, and cooks know how to handle frozen foods, and to cook them so as to guarantee they shall be placed on the table in prime condition for consumption. Frozen meat is often roasted or baked and, when cut into, presents, as the bone is approached, a blue appearance which shows that the heat has never penetrated to the bone. By obtaining one of Captain Warren's tins from the Stores, placing the chilled or frozen meat or fish in the tin, containing cold water, and allowing the water to be gradually heated until it approaches the boiling-point, the whole of the food in the tin becomes thoroughly thawed and ready, in the case of meat, to be taken from the tin, roasted for half an hour, and then served. When cut it will be found full of gravy, tender and delicious. In the case of fish, which of course takes much less time to cook, when the water simmers the fish should be ready to be taken out and served.

---

As Miss Macdonald, Matron-in-Chief of the Canadian Nursing Service, herself admitted, all her sisters "will want to be sick" in order that they may gain admittance to the new Rest House which was opened by Lady Perley on Monday last at 66 Ennismore Gardens, S.W. The house, rent free and furnished throughout, has been given by Colonel and Mrs. Gretton, and will accommodate thirty to forty sisters, who must be either convalescent from illness or tired out with war service abroad or in England. There is a spacious hall, a richly furnished drawing-room with oil paintings, its walls panelled and attractive with carved wood-work, a polished floor with thick rugs, and dainty little tables with bowls of flowers. There is also a grand piano. We were charmed by a fine old Grandfather clock and many inlaid tables and cabinets. There are, further, a cosy library provided with well-filled bookshelves and several writing desks, a dining-room and a snug reception-room on the first floor. All the bedrooms are attractive in their freshness and comfort. The sisters who sleep on the first floor will have a distinct advantage over those who occupy the top landing, but whether the furniture is elaborate or plainer, the whole of the rooms are fresh and inviting. Speaking generally, we may add that every arrangement has been made for the comfort of the guests, and convalescence at Ennismore Gardens should prove both grateful and enjoyable.

---

☛ IT is our invariable practice to pay for all items of news which we may publish. The minimum payment is 5s. Paragraphs of about twenty lines in length—that is to say, of 150 words or less—are preferred. Contributions should be addressed to The Editor, THE HOSPITAL, 28 and 29 Southampton Street, Strand, London, W.C. 2, and, to be in time for the current week's issue, should reach him by the first post on Mondays.

For Nursing Affairs in Ireland, see p. 336; Matrons' and Sisters' Appointments, see p. 344.

